# Preventing Dangerous Nonsense: Selection for Robustness to Transcriptional Error in Human Genes

**DOI:** 10.1371/journal.pgen.1002276

**Published:** 2011-10-13

**Authors:** Brian P. Cusack, Peter F. Arndt, Laurent Duret, Hugues Roest Crollius

**Affiliations:** 1 Max Planck Institute for Molecular Genetics, Department of Computational Molecular Biology, Berlin, Germany; 2 Institut de Biologie de l'Ecole Normale Supérieure (IBENS), CNRS UMR8197, INSERM U1024, Paris, France; 3 Université de Lyon, Université Lyon 1, CNRS, UMR 5558, Laboratoire de Biométrie et Biologie Evolutive, Villeurbanne, France; University of Michigan, United States of America

## Abstract

Nonsense Mediated Decay (NMD) degrades transcripts that contain a premature STOP codon resulting from mistranscription or missplicing. However NMD's surveillance of gene expression varies in efficiency both among and within human genes. Previous work has shown that the intron content of human genes is influenced by missplicing events invisible to NMD. Given the high rate of transcriptional errors in eukaryotes, we hypothesized that natural selection has promoted a dual strategy of “prevention and cure” to alleviate the problem of nonsense transcriptional errors. A prediction of this hypothesis is that NMD's inefficiency should leave a signature of “transcriptional robustness” in human gene sequences that reduces the frequency of nonsense transcriptional errors. For human genes we determined the usage of “fragile” codons, prone to mistranscription into STOP codons, relative to the usage of “robust” codons that do not generate nonsense errors. We observe that single-exon genes have evolved to become robust to mistranscription, because they show a significant tendency to avoid fragile codons relative to robust codons when compared to multi-exon genes. A similar depletion is evident in last exons of multi-exon genes. Histone genes are particularly depleted of fragile codons and thus highly robust to transcriptional errors. Finally, the protein products of single-exon genes show a strong tendency to avoid those amino acids that can only be encoded using fragile codons. Each of these observations can be attributed to NMD deficiency. Thus, in the human genome, wherever the “cure” for nonsense (i.e. NMD) is inefficient, there is increased reliance on the strategy of nonsense “prevention” (i.e. transcriptional robustness). This study shows that human genes are exposed to the deleterious influence of transcriptional errors. Moreover, it suggests that gene expression errors are an underestimated phenomenon, in molecular evolution in general and in selection for genomic robustness in particular.

## Author Summary

In biological systems mistakes are made constantly because the cellular machinery is complex and error-prone. Mistakes are made in copying DNA for transmission to offspring (“genetic mutations”) but are much more frequent in the day-to-day task of gene expression. Genetic mutations are heritable and therefore have long been the almost exclusive focus of evolutionary genetics research. In contrast, gene expression errors are not inherited and have tended to be disregarded in evolutionary studies. Here we show how human genes have evolved a mechanism to reduce the occurrence of a specific type of gene expression error—transcriptional errors that create premature STOP codons (so-called “nonsense errors”). Nonsense errors are potentially highly toxic for the cell, so natural selection has evolved a strategy called Nonsense Mediated Decay (NMD) to “cure” such errors. However this cure is inefficient. Here we describe how a preventative strategy of “transcriptional robustness” has evolved to decrease the frequency of nonsense errors. Moreover, these “prevention and cure” strategies are used interchangeably—the most transcriptionally robust genes are those for which NMD is most inefficient. Our work implies that gene expression errors play an important role as supporting actors to genetic mutations in molecular evolution, particularly in the evolution of robustness.

## Introduction

In mammalian transcripts, premature termination codons (PTCs) are normally detected by one of two NMD pathways [1]. The primary, and more efficient, NMD pathway is intron-dependent and relies on the presence of exon junction complexes (EJCs) deposited 20-24 nts upstream of the exon-exon junctions created following splicing of the mRNA (EJC-dependent NMD). A second, less efficient, intron-independent pathway operates in mammals and requires the presence of polyA-binding protein (PABP-dependent NMD). In contrast, in *Drosophila melanogaster*, PABP-dependent NMD is the primary pathway in operation since only a minority of spliced transcripts are under the surveillance of EJC-dependent NMD [2], [3]. The diversity of NMD mechanisms in eukaryotes is further illustrated among yeast species. In *Saccharomyces cerevisiae* only the intron-independent NMD pathway is known to operate. Finally, in *Schizosaccharomyces pombe*, although intron-dependent NMD is active it is believed to be EJC-independent [4].

PTCs may arise from heritable nonsense mutations in the germline or they can be created by transient errors in transcription and splicing. Previous attention has been paid to splicing errors as a source of nonsense errors since introns retained in the mature mRNA can either introduce an intron-encoded PTC or induce a frameshift leading to the formation of an exon-derived PTC [5]. However, mistranscription is likely to be a significant source of nonsense errors. Although few direct estimates are available, assuming a transcriptional error rate of 10^−5^ errors per nucleotide [6], [7] leads to the estimate that 0.05%-0.5% of transcripts of any given gene are expected to contain PTCs due to mistranscription [8]. Moreover, this is a highly conservative estimate since the single measurement available for a metazoan suggests that the rate of mistranscription may be as high as 10^−3^ errors per nucleotide [9].

The possible consequences of translating PTC-containing transcripts include loss of functional molecules, dominant negative interactions and gain-of-function activities. The deleterious effects of these outcomes are highlighted by the fact that, in mammals, knockouts of the core effectors of NMD have lethal effects [10].

NMD mitigates the negative effects of PTC-containing transcripts and hence can be considered as providing a partial “cure” for transcriptional errors. In mammals, the efficiency of NMD in detecting PTCs varies both among and within genes. Single-exon genes appear insensitive to NMD [11], [12] owing to the fact that these genes are served only by the inefficient PABP-dependent pathway and are invisible to the more potent EJC-dependent NMD pathway. As a result, PTC-bearing transcripts of single-exon genes are more likely to be translated into truncated protein species with toxic effects. Therefore, it follows that transcriptional errors creating nonsense codons are likely to be more deleterious in single-exon than in multi-exon genes. Equally, in multi-exon genes, the EJC-dependent NMD pathway is thought to only detect PTCs lying 50–55 nts upstream of the last exon-exon junction [13]. Consequently, like single-exon genes, last exons of multi-exon genes are invisible to EJC-dependent NMD in the mammalian genome and are served only by the PABP-dependent NMD pathway.

**Figure 1:**
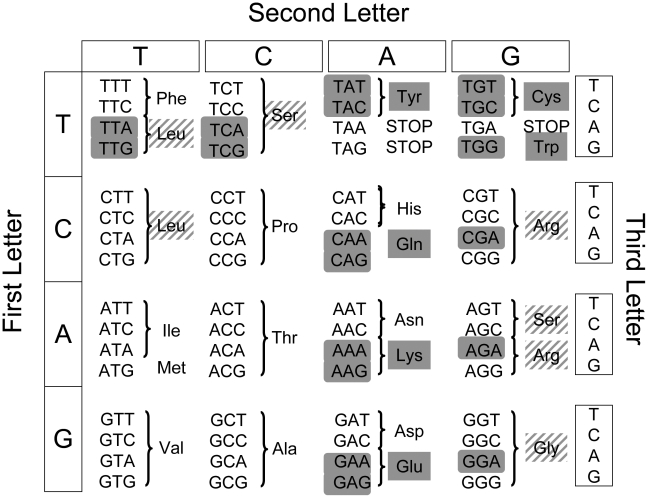
Sense codons differ in their propensity for conversion to STOP codons. The Standard Genetic Code contains 18 fragile codons (shaded) that can be changed into a STOP codon by a single point-mutation and whose mistranscription can therefore generate nonsense errors. The remaining 43 sense codons are “robust” to such errors. Six amino acids are encoded exclusively by fragile codons (“fragile amino acids”, shaded), ten amino acids are encoded exclusively by robust codons (“robust amino acids”, unshaded) and four amino acids can be encoded either by robust or fragile codons (“facultative amino acids”, hatched shading).

In this study we propose that natural selection has promoted a dual–strategy of prevention and cure to deal with nonsense errors in gene expression. Specifically we predict that selection compensates for the partial efficiency of NMD through a preventative approach of “transcriptional robustness” that minimizes the consequences of transcriptional error. We investigated whether human genes eschew codons that can be changed into a stop codon by one mutation (‘fragile codons’ [14]) in favour of codons robust to nonsense errors during mistranscription (‘robust codons’). The standard genetic code comprises 18 fragile codons and 43 robust codons (Figure 1). Of the 20 amino-acids, six are encoded exclusively by fragile codons (“fragile amino acids”), ten are encoded exclusively by robust codons (“robust amino acids”) and four can be encoded by codons of either type (“facultative amino acids”). This suggests two distinct mechanisms by which the transcriptional robustness of a gene can be increased by natural selection. First, at the level of synonymous codon usage fragile codons can be avoided when a robust synonym exists. Second, at the protein level fragile amino-acids can be counterselected.

Using comparisons between single and multi-exon genes (intergenic) and between different exons of multi-exons genes (intragenic), we show how each of these mechanisms is used to increase transcriptional robustness of mammalian genes. Single-exon genes should provide the clearest signal of transcriptional robustness and thus provide a “litmus test” of this hypothesis. Therefore, our first, intergenic, analysis contrasts fragile codon usage and fragile amino-acid usage in single-exon genes and multi-exon genes. We performed a second, intragenic, analysis at the exon-level by taking advantage of the fact that, in mammals, last exons of multi-exon genes, like single-exon genes, are invisible to EJC-dependent NMD. Finally, we demonstrate that the two hallmarks of transcriptional robustness detectable in mammals (synonymous codon usage and amino-acid usage) can also be detected in the genes of fission yeast (*Schizosaccharomyces pombe*) in which a mechanistically distinct form of intron-dependent Nonsense Mediated Decay appears to be active.

## Results/Discussion

### The inefficiency of NMD in single-exon genes is mitigated through the mechanism of synonymous codon choice

We used an intergenic comparison to focus initially on the first mechanism to achieving transcriptional robustness and quantified it using a normalized fragile codon usage (NFCU) metric. Being mutational neighbours of STOP codons, the 18 fragile codons are relatively AT-rich. It is known that GC-content varies along chromosomes and that these variations in base composition affect both synonymous codon usage and amino-acid usage. These variations are particularly strong within mammalian genomes (the so-called isochore genome organization). To account for both this compositional bias and the influence of amino acid content, we used codon usage among facultative amino acids as the basis of the NFCU metric. NFCU measures the relative usage of fragile and robust codons among 5 groups of codons where all codons in a given group (i) have the same GC-content and (ii) are synonymous.

We measured NFCU in 2422 single-exon genes and in 20563 multi-exon genes in the human genome. We observe a 8% depletion of fragile codons in single-exon genes that is highly significant (*p*<10^−15^, Wilcoxon rank sum test; Table 1). Similarly, in mouse, measuring NFCU in 3582 single-exon genes and 20263 multi-exon genes reveals a highly significant 11% depletion of fragile codons in single-exon genes. Both results provide preliminary support for our hypothesis. We performed a negative control by repeating the analysis in *D.melanogaster* in which both gene sets should on average be equally well served by NMD since the intron-dependent NMD pathway shows much reduced activity in fly [2], [3]. In the fly genome, the depletion of fragile codons in single-exon compared to multi-exon genes is almost null (2%) (Table 1) and, in contrast to human and mouse, can be explained by transcript length differences (see below).

**Table 1:** Normalized fragile codon content (NFCU) (controlling for GC-content and amino-acid usage) of multi-exon genes and single-exon genes in the human, mouse, and fly genomes.

		PABP-dependent NMD	EJC-dependent NMD	Genes	NFCU	*P*-value	Ratio
**Human genes**							
Multi-exon		**+**	**+**	20563	0.47		**1.00**
					(0.41–0.54)		
Single-exon:	All	**+/−**	**−**	2422	0.43	<10^−15^	**0.92**
					(0.34–0.51)		
	Non-histone	**+**	**−**	2367	0.43	<10^−15^	**0.92**
					(0.34–0.51)		
	Histone	**−**	**−**	55	0.32	<10^−9^	**0.68**
					(0.23–0.45)		
**Mouse genes**							
Multi-exon		**+**	**+**	20263	0.47		**1.00**
					(0.40–0.53)		
Single-exon:	All	**+/−**	**−**	3582	0.42	<10^−15^	**0.89**
					(0.33–0.51)		
	Non-histone	**+**	**−**	3533	0.42	<10^−15^	**0.89**
					(0.33–0.51)		
	Histone	**−**	**−**	49	0.22	<10^−15^	**0.47**
					(0.14–0.33)		
**Fly genes**							
Multi-exon		**+**	**−**	11643	0.54		**1.00**
					(0.48–0.60)		
Single-exon:	All	**+/−**	**−**	2498	0.53	0.0002	**0.98**
					(0.45–0.62)		
	Non-histone	**+**	**−**	2466	0.53	0.002	**0.98**
					(0.45–0.62)		
	Histone	**−**	**−**	32	0.38	<10^−9^	**0.69**
					(0.38–0.38)		

Single-exon genes were first treated as a single group to determine the impact of EJC-dependent NMD on fragile codon content and subsequently subdivided into histone and non-histone genes to consider the impact of PABP-dependent NMD on fragile codon content. NFCU, median (first and third quartiles) of fragile codon content normalized for GC-content and amino acid usage; *P*-value, significance of the comparison with multi-exon genes determined by two-sided Wilcoxon-rank sum test that tests for a linear shift in distribution locations; Ratio, median NFCU relative to the median NFCU for multi-exon genes.

Notably, repeating the analysis without controlling for amino acid usage or nucleotide composition but with the benefit of higher coverage of codons, yields essentially the same result (Text S1, Result A; Table S1).

### The invisibility of histone genes to NMD is mitigated by synonymous codon choice

Interestingly, one group of genes falls entirely outside of the range of NMD's surveillance. Replication-dependent histones contain neither introns in their coding sequences nor polyA-tail in their mRNAs [15]. Therefore, histone genes represent a blind-spot for both mammalian NMD pathways [12]. According to our hypothesis histone genes should represent the most transcriptionally robust genes in the mammalian genome since PTC-containing transcripts of their genes will not be recognized and degraded before translation. In agreement with this, in human we see that NFCU in histone genes is 32% lower than that of multi-exon genes (Table 1) and 26% lower than that of other single-exon genes. Similarly, in mouse we see that NFCU in histone genes is 53% lower than that of multi-exon genes (Table 1) and 47% lower than that of other single-exon genes. Notably, we found that histone genes in fly show a 30% depletion of NFCU compared to other single exon genes in accordance with the fact that histone genes are not served by NMD in fly (Table 1).

### The inefficiency of NMD in single-exon genes is also mitigated through the mechanism of amino-acid choice

The analysis of fragile codon usage controlling for GC and amino-acid usage (NFCU) shows that the depletion of fragile codons in human and mouse single-exon genes is not a consequence of amino acid usage. Therefore, we can ask whether there is evidence for usage of the second mechanism of transcriptional robustness by testing whether the amino acid usage of single-exon gene products reduces the frequency of nonsense transcriptional errors. Accordingly, we observed that the usage of fragile amino acids (FAU) among proteins encoded by single-exon genes is 17% lower than those of multi-exon genes in human and 21% lower in mouse whereas no difference is observed in fly (Table 2). Therefore the constraints on codon usage in human genes imposed by the need for transcriptional robustness appear to be strong enough to influence their amino-acid sequences.

**Table 2:** Fragile amino acid usage (FAU) of proteins encoded by multi-exon genes and single-exon genes in the human, mouse, and fly genomes.

	PABP-dependent NMD	EJC-dependent NMD	Genes	FAU	*P*-value	Ratio
**Human genes**						
Multi-exon	**+**	**+**	20573	0.23		**1.00**
				(0.20–0.27)		
Single-exon:	**+/−**	**−**	2424	0.19	<10^−15^	**0.83**
				(0.16–0.24)		
**Mouse genes**						
Multi-exon	**+**	**+**	20284	0.24		**1.00**
				(0.21–0.27)		
Single-exon:	**+/−**	**−**	3589	0.19	<10^−15^	**0.79**
				(0.16–0.24)		
**Fly genes**						
Multi-exon	**+**	**−**	11643	0.23		**1.00**
				(0.20–0.26)		
Single-exon:	**+/−**	**−**	2498	0.23	0.97	**1.00**
				(0.20–0.26)		

FAU, median (first and third quartiles) of fragile amino acid content of encoded proteins; *P*-value, significance of the comparison with multi-exon genes determined by two-sided Wilcoxon-rank sum test that tests for a linear shift in distribution locations; Ratio, median FAU relative to the median FAU for multi-exon genes.

We repeated this analysis to control for GC content differences between single-exon and multi-exon genes. We used a normalized fragile amino-acid usage metric (NFAU) that considers the relative usage of fragile amino-acids among two groups of amino-acids that are encoded by codons having the same GC-content. Once GC content is controlled for we observe that the fragile amino acid content of single-exon gene products is 12% lower than those of multi-exon genes in human and 16% lower in mouse whereas a 2% enrichment is seen in fly (Table 3).

**Table 3:** Normalized fragile amino acid usage (NFAU) (controlling for GC-content) of proteins encoded by multi-exon genes and single-exon genes in the human, mouse, and fly genomes.

	PABP-dependent NMD	EJC-dependent NMD	Genes	NFAU	*P*-value	Ratio
**Human genes**						
Multi-exon	**+**	**+**	20573	0.44		**1.00**
				(0.39 – 0.48)		
Single-exon	**+/−**	**-**	2424	0.38	<10^−15^	**0.88**
				(0.32 – 0.47)		
**Mouse genes**						
Multi-exon	**+**	**+**	20284	0.43		**1.00**
				(0.39 – 0.48)		
Single-exon	**+/−**	**-**	3588	0.36	<10^−15^	**0.84**
				(0.31 – 0.46)		
**Fly genes**						
Multi-exon	**+**	**−**	11643	0.41		**1.00**
				(0.37 – 0.46)		
Single-exon	**+/−**	**−**	2498	0.42	<10^−4^	**1.02**
				(0.37 – 0.48)		

NFAU, median (first and third quartiles) of normalized fragile amino acid content of encoded proteins; *P*-value, significance of the comparison with multi-exon genes determined by two-sided Wilcoxon-rank sum test that tests for a linear shift in distribution locations; Ratio, median NFAU relative to the median NFAU for multi-exon genes.

### The correlation between both transcriptional robustness mechanisms is dependent on selective constraint

Thus far our analysis has demonstrated that, in human and mouse, single exon genes bear two hallmarks of transcriptional robustness relative to multi-exon genes (i.e. depletion of fragile codons and amino acids). These hallmarks highlight two mechanisms by which natural selection can prevent nonsense transcriptional errors: at the level of gene sequence through synonymous codon choice and at the level of protein sequence through amino-acid choice. Provisionally, we can attribute this observation to the inactivity of EJC-dependent NMD in single-exon genes. However, since all biological processes are inherently inefficient, EJC-dependent NMD is inevitably also suboptimal. This raises the question whether there is a genome-wide requirement for transcriptional robustness. One approach to answering this question is to ask whether, among all human genes, there is correlated usage of the two mechanisms (codon-level and protein-level) to achieving transcriptional robustness.

However, for a given gene the nature of the correlation between these two mechanisms is likely to depend on the relative level of constraint on synonymous and non-synonymous sites as measured by K_a_/K_s_ (the ratio of non-synonymous to synonymous substitution rates). For the vast majority of human genes, estimates of K_a_/K_s_ lie in the range 0-1. Broadly, we might expect two different patterns: (i) For genes with K_a_/K_s_ close to 1, synonymous and non-synonymous sites are equally modifiable. Therefore, where nonsense errors are deleterious, natural selection can use both mechanisms to increase transcriptional robustness (i.e. both fragile codons and fragile amino-acids can be depleted) leading us to expect an overall positive correlation between fragile codon usage and fragile amino-acid usage. (ii) For genes with K_a_/K_s_ close to 0 there is strong selective constraint on the protein sequence and non-synonymous sites are much less modifiable than synonymous sites. Here natural selection can use only one mechanism to increase transcriptional robustness: that of synonymous codon choice since transcriptional robustness can be less readily increased through amino-acid choice. Notably, the protein products of many such genes might be enriched in fragile amino acids due to functional requirements e.g. proteins involved in signal-transduction tend to be tyrosine rich and zinc-finger proteins and proteins enriched in disulfide bonds are cysteine rich. If nonsense errors are deleterious in such genes then there should be strong counterselection of fragile codons to compensate for functionally-determined fragile amino-acid content. For genes under strong selective constraint at the amino-acid level we would therefore expect an overall negative correlation between fragile codon usage and fragile amino-acid usage. Among genes with strong selective constraint on protein sequence, the magnitude of this negative correlation is likely to depend on the abundance of genes with functions requiring high fragile amino-acid content.

**Figure 2:**
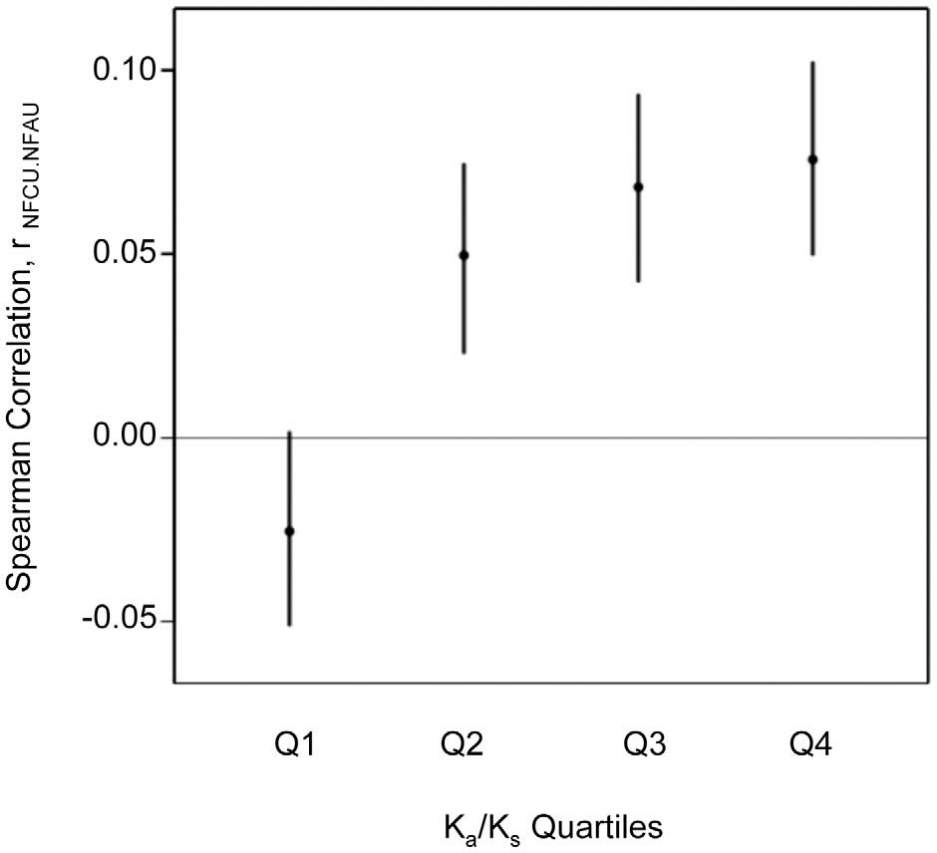
The genome-wide correlation between transcriptional robustness strategies depends on selective constraint. Pairwise correlation between normalized fragile codon usage (NFCU) and normalized fragile amino-acid usage (NFAU) for 17421 human genes with an ortholog in mouse. Human genes were binned by selective constraint (K_a_/K_s_) estimated using the pairwise alignment with their mouse ortholog and for each quartile of K_a_/K_s_ (Q1, lowest, to Q4, highest), Spearman's correlation between normalized fragile codon usage and normalized fragile amino-acid usage (r_NFCU.NFAU_) was calculated. The vertical extent of the bar indicates the 95% confidence interval for each correlation.

Considering all human genes (single-exon and multi-exon genes) we see a positive correlation between both transcriptional robustness mechanisms (Spearman correlation for NFCU versus NFAU: rho = 0.06, n = 22985, *p*<10^−15^). More specifically, for human genes having mouse orthologs we can determine how this correlation depends on the strength of selective constraint. We observe that the sign and magnitude of the correlation between both transcriptional robustness mechanisms (codon-level and protein-level) is dependent on the strength of selective constraint on protein sequence in agreement with our prediction (Figure 2).

First, for genes towards the upper end of the K_a_/K_s_ range, our first prediction holds. Thus among genes under weaker selective constraint at the protein level (mean K_a_/K_s_ for the fourth quartile of selective constraint  =  0.38), we observe a positive correlation between normalized fragile codon usage (NFCU) and normalized fragile amino-acid usage (NFAU) (Figure 2). In other words, for genes in which non-synonymous sites are, on average, only 60% less modifiable than synonymous sites, both transcriptional robustness mechanisms are available to selection. Moreover, it is striking that among the top three quartiles of K_a_/K_s_ the positive correlation between both mechanisms increases in magnitude with weakening selective constraint (Spearman's rho (p-values) for NFCU versus NFAU in K_a_/K_s_ quartiles 2-4: 0.050 (*p* = 0.001); 0.068 (*p*<10^−5^); 0.076 (*p*<10^−6^)). Therefore with increasing ‘flexibility’ of the protein sequence there is an increasing tendency for transcriptional robustness to be realized not only through synonymous codon choice but also through amino-acid choice.

However, for genes under strong selective constraint at the protein level (mean K_a_/K_s_ for the first quartile of selective constraint  =  0.03) the correlation between transcriptional robustness mechanisms is negative (Spearman's rho for NFCU versus NFAU in K_a_/K_s_ quartile 1: -0.025 (*p* = 0.09); Figure 2). For such genes non-synonymous sites are, on average, 97% less modifiable than synonymous sites and a requirement for transcriptional robustness can only be accommodated at the level of synonymous codon usage and not at the level of amino-acid usage.

In summary, the fact that the genome-wide correlation between usage of fragile codons and fragile amino-acids depends on selective constraint points to a universal requirement for transcriptional robustness to nonsense errors among human genes.

### The absence of splicing constraints cannot explain the lower fragile codon content of single-exon genes

A simple interpretation of the lower fragile codon content of single-exon genes is that this observation may be due to differences in splicing-related constraints between single and multi-exon genes. Splicing requires regulatory sequences located in exons known as exonic splicing enhancers (ESEs). Since these hexamer sequences overlap codons, their presence imposes additional constraints on the coding sequence of multi-exon genes [16] in contrast to the coding sequence of single-exon genes that have no such splicing constraints. Therefore, if the nucleotide composition of ESEs is such that they tend to encode fragile codons then a simple difference in ESE density between single and multi-exon genes could provide a trivial explanation for the relative enrichment of fragile codons in the latter group.

We found that ESEs indeed tend to encode fragile codons and that ESEs are depleted in single-exon genes. However the difference in fragile codon usage between single and multi-exon genes persists when this is controlled for showing that our observation is not a side-effect of differences in splicing related constraints (Text S1, Result B; Figure S1).

### The lower fragile codon content of single-exon genes cannot be explained by selection for translationally optimal codons

The patterns of fragile codon usage in single-exon and multi-exon genes could be due to a more familiar source of codon usage bias that has nothing to do with their propensity for nonsense errors. In fly, selection for translational efficiency or accuracy leads to the preferential usage of optimal codons in highly expressed genes [17]. Moreover, recent evidence suggests that such selection might also operate on mammalian genes [18]–[20]. The possible influence of selection for translational efficiency raises two potential concerns for our analysis. First, in human, it might create an artifactual difference in fragile codon usage of the magnitude we observe between single and multi-exon genes leading us to falsely infer a difference in transcriptional robustness between these gene sets. Second, in fly, it might obscure a real difference in transcriptional robustness by homogenizing fragile codon usage between single and multi-exon genes and therefore might invalidate the use of the *Drosophila* genome as a negative control.

Among codons we see no association in either human or fly between fragility with respect to nonsense errors and translational optimality (see Text S1, Result C). Nevertheless, to account for the possibility that there is an association among genes between transcriptional robustness and selection for translational accuracy we repeated the analysis of fragile codon content in human and fly and controlled for the fraction of optimal codons per gene (F_op_) [17], [21].

Comparing human single and multi-exon genes binned in this way revealed that the difference in both FCU and NFCU persists independently of their optimal codon usage (see Text S1, Result C; Figure S2). Thus in human, the difference in fragile codon usage between single and multi-exon genes is not an artifact of selection for translationally optimal codon use. Additionally, in fly, the control for optimal codon usage has no influence on the magnitude of the difference between single and multi-exon genes with respect to either FCU or NFCU (see Text S1, Result C; Figure S3). Thus in fly, selection for translationally optimal codons does not mute any signal for transcriptional robustness among single-exon genes.

### Intragenic comparison shows that fragile codons are depleted in last exons of human multi-exon genes

The fact that only PTCs lying more than ∼50–55 nts upstream of the last Exon Junction Complex (EJC) are thought to be detected by the EJC-dependent NMD pathway suggests that, similarly to single-exon genes, last exons of multi-exon genes constitute a blind-spot for EJC-dependent NMD and are served only by PABP-dependent NMD. Although a mechanism for detection of PTCs downstream of the last EJC has been described (“fail-safe” NMD), its activity is believed to be restricted to exceptional targets [22]. We therefore hypothesized that last exons of multi-exon genes should show a depletion of fragile codons comparable to that seen in single-exon genes.

For human multi-exon genes whose coding sequence is completely contained within the last exon we see a significant 8% depletion of fragile codons compared to multi-exon genes having at least two coding exons (see Text S1, Result D). We next used an intragenic comparison to ask whether the last exons of all human multi-exon genes show a similar depletion of fragile codons. To address this we performed an exon-based analysis of human multi-exon genes that have a reliably annotated last exon and at least one upstream coding exon. We created one group of last exons and one group of upstream exons and compared these with respect to NFCU. We found that fragile codons exhibited a significant (*p* = 0.0002, Wilcoxon rank-sum test) 7% depletion among last exons (n = 12390, median NFCU = 0.47) compared to upstream exons (n = 112789, median NFCU = 0.50) (Table 4). We repeated the analysis in mouse and observed a 8% depletion of fragile codons (*p* = 0.002, Wilcoxon rank-sum test) among last exons (n = 13265, median NFCU = 0.46) compared to upstream exons (n = 132678, median NFCU = 0.50). As a control we performed the same analysis on *Drosophila* multi-exon genes in which NMD should be equally efficient in upstream and last exons. In accordance with our expectation we saw no depletion of fragile codons among last exons (n = 6304, median NFCU = 0.53) compared to upstream exons (n = 25686, median NFCU = 0.53) of *Drosophila* multi-exon genes.

**Table 4:** Normalized fragile codon usage (NFCU) of last exons and upstream exons of multi-exon genes in the human, mouse, and fly genomes.

		PABP-dependent NMD	EJC-dependent NMD	Exons	NFCU	*P*-value	Ratio
Human multi-exon genes							
Upstream exons		**+**	**+**	112789	0.50		**1.00**
					(0.39 – 0.48)		
Last exons		**+**	**−**	12390	0.47	0.0002	**0.93**
					(0.29 – 0.61)		
Mouse multi-exon genes							
Upstream exons		**+**	**+**	132678	0.50		**1.00**
					(0.26 – 0.67)		
Last exons		**+**	**−**	13265	0.46	0.002	**0.92**
					(0.30 – 0.60)		
Fly multi-exon genes							
Upstream exons		**+**	**−**	25686	0.53		**1.00**
					(0.41 – 0.67)		
Last exons		**+**	**−**	6304	0.53	0.21	**1.00**
					(0.41 – 0.67)		

NFCU, median (first and third quartiles) of fragile codon content normalized for GC-content and amino acid usage; *P*-value, significance of the comparison of upstream and last exons determined by two-sided Wilcoxon-rank sum test that tests for a linear shift in distribution locations; Ratio, median NFCU relative to the median NFCU for upstream exons.

These results were confirmed by an analysis based on matching the last exon of each human multi-exon gene with the upstream sequence of the same gene. There are 12324 human multi-exon genes with reliably annotated last exons and for which NFCU is defined in both the last exon and in the upstream sequence. Among the 12265 genes for which NFCU differs between these two regions, for 6599 (54%) fragile codon content is lower in the last exon than in the upstream sequence whereas in 5666 genes NFCU in the last exon is greater than in the upstream sequence (*p*<10^−16^, binomial test). Considering NFCU for all 12324 pairs of upstream and last exons showed that their fragile codon content is significantly different (*p*<10^−15^, Wilcoxon signed-rank test).

Notably, we found that the depletion of fragile codons in last exons of human multi-exon genes can not be explained by the different splicing requirements for the final exon of transcripts [23] (see Text S1, Result E).

### Intragenic depletion of fragile codons commences beyond the boundary of EJC–dependent NMD activity

The depletion of fragile codons in last exons is particularly striking given the fact that a reduction in the selective costs of premature peptide truncation in last exons might mute any signal of transcriptional robustness caused by inefficient NMD in these exons. In other words, if NMD was equally efficient in upstream and last exons we would expect fragile codons to be enriched in last exons because PTCs generated by mistranscription in last exons are, on average, closer to the normal termination codon (NTC). If these PTCs remain undetected by NMD, the resultant short peptide truncations should have trivial fitness consequences compared to longer peptide truncations caused by undetected PTCs in upstream exons. Consistent with this, the fragile codon content of last exons of human multi-exon genes (median NFCU = 0.47) is greater than that of human single-exon genes (median NFCU = 0.43) despite the fact that both are invisible to EJC-mediated NMD.

**Figure 3:**
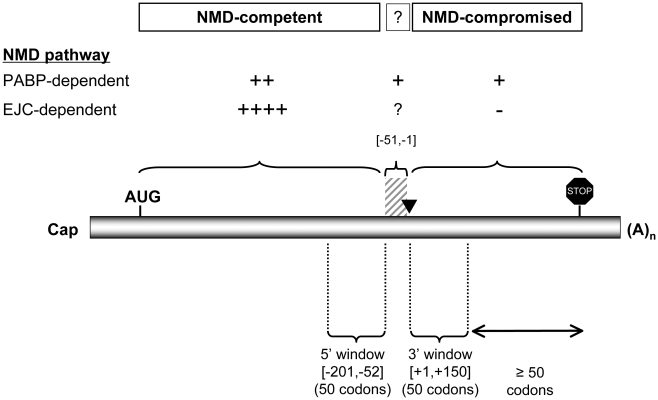
Intragenic depletion of fragile codons commences beyond the boundary of EJC–dependent NMD activity. Normalized fragile codon usage (NFCU) in “NMD-competent” and “NMD-compromised” regions of multi-exon genes. The schematic depicts a generic mammalian mature mRNA. The arrowhead shows the position of the last exon-exon junction. The relative efficiency of each mammalian NMD pathway predicted in three distinct regions is shown using ‘+’ symbols. Predicted inactivity of NMD is shown using a ‘-’ symbol. EJC-dependent NMD is expected to be active >50–55 nts 5′ of the last exon-exon junction and inactive 3′ of the last exon-exon junction. Its activity is uncertain in the intervening 50–55 nts region (hatched shading). PABP-dependent NMD is expected to increase in efficiency with distance from the poly-A tail. Note that the efficiency of PABP-dependent NMD is predicted to be much lower than that of EJC-dependent NMD. NFCU was determined in two windows of 50 codons positioned on either side of the last exon-exon junction. The example shows the last intron in “phase 0” (i.e. the intron is positioned between codons) and the depicted nucleotide coordinates for each window are specific to this case.

We attempted to control for the effect of trivial peptide truncation by analyzing NFCU in regions on either side of the boundary of EJC-dependent NMD but located at least 50 codons away from the normal termination codon. We focused on multi-exon genes having reliably annotated last exons longer than 100 codons and having at least 50 codons in the NMD-competent region of upstream exons (Figure 3). Specifically, for each multi-exon gene we calculated NFCU in a 3′ window comprising the first 50 codons encoded by the last exon and contrasted this with NFCU calculated from a neighbouring 50-codon 5′ window ending 50 nts upstream of the last exon-exon junction. For human multi-exon genes the 3′ window lies in the NMD-compromised region (invisible to EJC-dependent NMD) and the 5′ window lies in the NMD-competent region (visible to EJC-dependent NMD). The boundary between NMD-competent and NMD-compromised regions of human multi-exon genes is described as lying 50–55 nts upstream of the last exon-exon junction (the “50- to 55- nts rule”) but the universality of this rule is unclear in the light of more recent genome-wide observations [24] and individual gene studies [25]. Consequently, the parameter settings for positioning these windows were chosen to reflect the uncertainty in the position of this boundary.

In this analysis we saw a 10% reduction in fragile codon density in 3′ (NMD-compromised) windows compared to 5′ (NMD-competent) windows (n = 2616; 5′ windows, median NFCU = 0.50; 3′ windows, median NFCU = 0.45; *p* = 0.016, Wilcoxon rank-sum test). As a negative control we repeated this analysis on *Drosophila* multi-exon genes in which EJC-dependent NMD shows reduced activity implying that surveillance of nonsense errors should be equally efficient in the 5′ and 3′ windows. As expected we saw no difference in fragile codon density between these window sets (n = 2420; 5′ windows, median NFCU = 0.5; 3′ windows, median NFCU = 0.5; *p* = 0.199, Wilcoxon rank-sum test).

We repeated this test to account for the reduced density of ESEs in human last exons by partitioning the codons in each window into those that overlap ESEs and those external to ESEs and then recalculating NFCU. Both analyses qualitatively agreed with the full analysis considering all 50 codons in each window (data not shown).

In summary, we see a consistent pattern of depletion of fragile codons in the last exons of human multi-exon genes when compared with upstream exons. This accords with their status as NMD-compromised regions that are invisible to EJC-dependent NMD and visible only to the PABP-dependent NMD pathway. The selective pressure to avoid fragile codons in last exons imposed by the requirement for transcriptional robustness is likely to be partly offset by increased tolerance of PTCs lying close to the NTC. Nevertheless, transcriptional robustness appears to be an important determinant of codon choice in the last exons of human multi-exon genes as well as in single-exon genes.

### Depletion of fragile codons is due primarily to inactivity of EJC–dependent NMD but also to reduced efficiency of PABP–dependent NMD

Since transcriptional robustness is likely to be a consequence of reduced NMD potency we can attempt to dissect the relative contributions to this phenomenon of each of the two mammalian NMD pathways. The large depletion of fragile codons in histone genes relative to other single-exon genes (Table 1) suggests that much of the variation in transcriptional robustness among and within human genes might be explained by variation in the efficiency of PABP-dependent NMD. Notably, the ability of this pathway to detect PTCs increases with the distance between the PTC and the polyA-binding protein (PABP) [1]. Single-exon genes produce shorter mRNAs than multi-exon genes (the coding sequences (CDS) of human single-exon genes and multi-exon genes have a median length of 534 nts and 1203 nts, respectively). Multi-exon genes may therefore be subject to more potent NMD than single-exon genes owing simply to more efficient surveillance by the PABP-dependent NMD pathway. This follows from the fact that, in multi-exon genes, any PTCs formed by mistranscription will be, on average, more distant from the PABP and thus will elicit PABP-dependent NMD more efficiently.

**Figure 4:**
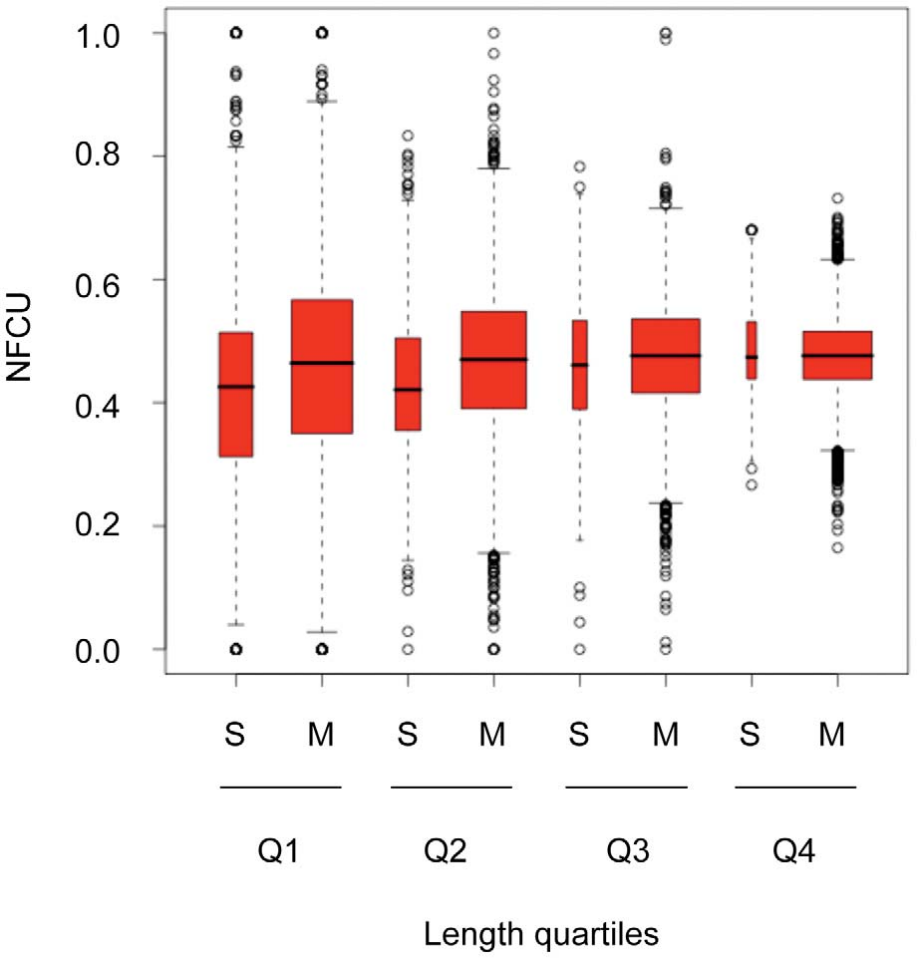
Fragile codon depletion is not due to reduced efficiency of PABP–dependent NMD in shorter mRNAs. Normalized fragile codon usage (NFCU) of human single- (S) and multi-exon (M) genes binned by transcript CDS length. For each quartile of transcript length (Q1, shortest, to Q4, longest) NFCU for single and multi-exon genes is plotted separately. The width of each bin is proportional to the square root of the number of genes it contains.

We investigated whether decreased efficiency of PABP-dependent NMD alone is responsible for the greater transcriptional robustness of single-exon genes due to the shorter length of their transcripts. We compared NFCU between single and multi-exon genes binned by CDS length (Figure 4) and found that NFCU was significantly lower in single exon genes than in multi-exon genes for all length bins except the longest (Q4): the ratios (p-values; Wilcoxon-rank sum test) of median NFCU in single-exon genes relative to median NFCU in multi-exon genes for length bins Q1-Q4 are 0.92 (*p*<10^−15^), 0.90 (*p*<10^−15^), 0.97 (*p* = 0.03), 0.99 (*p* = 0.91), respectively. Thus only the longest single-exon transcripts have fragile codon content equal to that of multi-exon transcripts of similar length. This result suggests that the increased transcriptional robustness of single-exon genes is primarily due to the lack of supplementary nonsense surveillance from EJC-dependent NMD. However, the longest single-exon transcripts also benefit from an increase in the efficiency of PABP-dependent NMD.

Repeating this procedure in our analysis of fly genes revealed no difference in NFCU between single-exon and multi-exon genes when length differences are controlled for (data not shown). Thus the modest 2% depletion of fragile codons seen in fly single-exon genes compared to multi-exon genes is entirely due to the inefficiency of PABP-dependent NMD of shorter transcripts (Table 1).

### Transcriptional robustness is also a property of genes that are invisible to splicing-dependent NMD in fission yeast

It has recently been demonstrated that splicing enhances NMD in *Schizosaccharomyces pombe* in a manner apparently independent of the EJC [26]. This organism provides us with an independent test of our hypothesis in a system of splicing-dependent NMD that is mechanistically distinct from that in mammals. We measured NFCU in *S.pombe* genes and found that single-exon genes (median NFCU = 0.43) show a highly significant (*p*<10^−15^; Wilcoxon-rank sum test) 6% depletion of fragile codons relative to multi-exon genes (median NFCU = 0.46) echoing our observations in human and mouse. However, in contrast to the situation in mammals and fly, the CDS of single-exon genes is longer than that of multi-exon genes in *S.pombe* (the coding sequence (CDS) of *S.pombe* single-exon genes and multi-exon genes have a median length of 1220 nts and 1053 nts, respectively). Therefore we tested for the possibility that the signal of robustness in single-exon genes in *S.pombe* is muted by the reduced efficiency of PABP-dependent NMD in the shorter mRNAs of multi-exon genes. We compared single and multi-exon genes in bins of equal CDS length and found that fragile codons are significantly depleted in single-exon genes for all length bins except the shortest: the ratios (p-values; Wilcoxon-rank sum test) of median NFCU in single-exon genes relative to median NFCU in multi-exon genes for length bins Q1-Q4 are 0.99 (*p* = 0.83), 0.92 (*p*<10^−7^), 0.93 (*p*<10^−8^), 0.95 (*p*<10^−8^), respectively).

The signal associated with the second mechanism of transcriptional robustness (amino-acid choice) is less strong in *S.pombe.* Among fission yeast proteins we observe a 3% depletion (*p*<10^−14^; Wilcoxon-rank sum test) of fragile amino-acids in the protein products of single-exon genes (median NFAU = 0.397) compared to multi-exon gene products (median NFAU = 0.410).

### Conclusion

In this study we set out to investigate whether human genes have evolved “transcriptional robustness” to reduce the frequency of mistranscription events leading to nonsense errors. More specifically we hypothesized that such a strategy of nonsense prevention should complement the cure for nonsense errors provided by Nonsense Mediated Decay (NMD). Indeed by comparing single-exon genes (in which NMD is inefficient) and multi-exon genes (in which NMD is much more efficient) we show that intergenic variation in transcriptional robustness reflects intergenic differences in NMD efficiency. Specifically, the primary hallmark of this robustness is a depletion of “fragile” codons that are susceptible to mistranscription into a STOP codon. We took account of differences between single-exon and multi-exon genes that, although unrelated to transcriptional robustness, might nevertheless covary with fragile codon usage. Our result is not explained by biologically confounding variables such as differences in splicing constraints or transcription associated mutational biases (see Text S1, Result F) nor by possible technical biases due to gene annotation issues or phylogenetic dependencies (see Text S1, Result G). Having excluded these alternatives we can conclude that, in human, there is a real difference in transcriptional robustness of single-exon and multi-exon genes.

Is there an alternative explanation for intergenic variation in transcriptional robustness that is unrelated to the efficiency of NMD? Before attributing our observation to intergenic differences in NMD efficiency, other differences between single-exon and multi-exon genes that could promote robustness need to be considered. Notably our intergenic analysis makes two assumptions: all genes have (i) equal transcriptional error rates and (ii) similar selective costs associated with the toxic effects of truncated proteins. However, if either of these quantities was, on average, greater in single-exon genes than in multi-exon genes then this would promote increased transcriptional robustness of single-exon genes even if NMD were equally potent in both groups.

Our analysis of intragenic patterns of fragile codon use addresses both assumptions, by isolating the influence on transcriptional robustness of variable NMD efficiency from the influences of variable transcriptional fidelity and selective costs of nonsense errors. First, although transcriptional fidelity might vary between genes, it is not likely to vary within genes. In contrast, the efficiency of NMD varies not only between genes but also within genes since, like single-exon genes, last exons of multi-exon genes are invisible to EJC-dependent NMD in mammals. Therefore if selection for transcriptional robustness was mediated by transcriptional fidelity then this could explain the intergenic but not the intragenic patterns of fragile codon use that we see (i.e. depletion of fragile codons in last exons). Second, although the selective costs of nonsense errors might vary within genes this should result in an enrichment of fragile codons in last exons rather than the depletion observed.

Therefore the intragenic pattern of fragile codon usage in mammalian genes, and in particular the depletion of fragile codons in last exons, suggests that variable NMD-efficiency underlies transcriptional robustness. Although these patterns do not implicate variation in transcriptional fidelity or in the selective costs of undetected nonsense errors as unique explanations of transcriptional robustness these factors might also contribute to intergenic differences in robustness. Importantly, the result of our intragenic analysis strongly suggests that constraints on codon usage imposed by the requirement for transcriptional robustness are common to all mammalian genes and are not peculiar to the minority of genes that are intronless in mammals (∼11% and ∼15% of genes in the human and mouse genomes, respectively).

We conclude therefore, that natural selection has promoted a dual strategy of “prevention and cure” to deal with the problem of nonsense transcriptional errors and that these strategies are used interchangeably in mammals. This example of complementarity between strategies for error-prevention and error-mitigation in mammals echoes the recent demonstration in bacteria of complementarity between *cis* and *trans* strategies in limiting protein misfolding [27].

There is evidence that both the intron content [5] and the exon-intron structure [24], [28], [29] of human genes are shaped by the general mode of action and specific spatial requirements of NMD. We show that variable NMD efficiency also leaves its signature in the coding sequences of human genes and in the amino-acid content of the proteins they encode. This signature can be discerned in patterns of codon choice and amino-acid usage in single-exon genes that together constitute two hallmarks of transcriptional robustness. It is also evident in codon usage in the last exons of multi-exon genes and thus constrains a substantial fraction of sites in human genes. We suggest that fragile codons are counterselected in human genes not because they pose a potential future mutational hazard but because of the immediate hazard associated with undetected nonsense errors during their transcription. However, a notable side-effect of transcriptional robustness is the creation of a “congruent robustness” [30] to future nonsense-creating genetic mutations. The negative selection invoked here is mediated by transcriptional errors and not by genetic mutations. Indeed, transcriptional errors, together with splicing and translation errors, may exert a negative effect on fitness despite the presence of a normal genotype. Such errors in gene expression [31], [32] may play a much more prominent role in molecular evolution than is currently recognized [33]. One such role may be to expose subtle fitness differences between otherwise equally-fit genotypes, enabling positive selection to explore sequence space in the vicinity of a given genotype by means of a so-called “look-ahead effect” [33]. Equally, we suggest, the evolutionary foresight gained from this effect may reveal pitfalls in sequence space and enable negative selection to purge genotypes that lie close to these pitfalls by providing a preview of their deleterious consequences. This effect provides a rationale for the promotion of robustness by natural selection even when the population genetic conditions of high genetic mutation rate and large effective population size, conventionally thought to be necessary for the evolution of robustness [34], are not met. Together with the identification of robustness to translational errors [18] and to splicing errors [5], this study underscores the importance in molecular evolution of the full spectrum of errors made in the decoding of phenotype from genotype. Moreover, the patterns of fragile codon usage and fragile amino-acid usage described for human genes suggest that transcriptional errors are frequent and can be highly deleterious. This raises the question of the past and present impact of such errors on human disease.

## Materials and Methods

### Datasets

We used Ensembl release 49 gene annotations for human and mouse and Flybase release 5.4 for *Drosophila* annotations [35]. For each gene prediction we retrieved its CDS from Ensembl. In the case of multiple alternative transcript predictions, we retained the transcript encoding the longest peptide and used the total number of annotated coding and non-coding exons in the transcript as the exon count for that gene. A dataset of histone genes was constructed by retrieving 72 Genbank accessions for human histone genes from [15] and mapping these to unique Ensembl gene identifiers using the BioMart tool. Fly orthologs of human histone genes were retrieved using BioMart. We constructed a dataset of exonic splicing enhancers (ESEs) consisting of 443 hexamer sequences by merging human and mouse ESEs determined using the RESCUE-ESE approach [36]-[38].

### Fragile codon usage (FCU)

We defined two categories of codons using a classification introduced by [14] : “fragile codons” are defined as sense codons that can be converted into a STOP codon by a single point-mutation whereas all other sense codons are defined as “robust” (Figure 1). The fragile codon usage (FCU) metric considers all 61 sense codons and was calculated by enumerating all fragile and robust codons and expressing fragile codon density (FCU) as the fraction of all sense codons that are fragile for each gene.

### Normalized fragile codon usage (NFCU)

Normalized fragile codon usage (NFCU) was calculated by considering only groups of codons that are synonymous and have equal GC content but differ with respect to their fragility. These groups were chosen from among codons encoding “facultative amino acids” (Figure 1). Four such groups have two-members that are respectively fragile and robust: TCA, TCT (encoding Serine; 1/3 nts are G or C), TCG, TCC (encoding Serine; 2/3 nts are G or C), CGA, CGT (encoding Arginine; 2/3 nts are G or C) and GGA, GGT (encoding Glycine; 2/3 nts are G or C). A fifth, three membered, group encodes Lysine and consists of TTG, CTT and CTA (fragile, robust and robust codons respectively; 1/3 nts are G or C). For each CDS we computed the fractional fragile codon usage for each of these five synonymous groups (number of fragile codons/the total number of codons in the group). Finally, for each gene we expressed the normalized fragile codon usage (NFCU) of its CDS as the average of all fractions that are defined (i.e. that have a denominator greater than zero).

### Fragile amino-acid usage (FAU)

For each gene we considered its encoded peptide (using the longest in the case of alternative isoforms) and calculated the fraction of all amino acids that are fragile (Cys, Gln, Glu, Lys, Trp, Tyr) (Figure 1) considering all amino-acids for each protein (i.e. FAU  =  count of fragile amino-acids/total count of amino-acids).

### Normalized fragile amino-acid usage (NFAU)

Normalized fragile amino-acid usage (NFAU) was calculated by considering only groups of amino-acids that are encoded by codons of equal GC content. For each gene we considered its longest encoded peptide and, for two separate groups of amino-acids, calculated the fraction of amino-acids that are fragile. The first group consists of four amino-acids encoded by codons for which 1/6 nts are G or C: Tyr (fragile), Lys (fragile), Asn (robust) and Phe (robust). The second group consists of eight amino-acids encoded by codons for which 1/2 nts are G or C: Gln (fragile), Glu (fragile), Cys (fragile), Ser (facultative), His (robust), Thr (robust), Val (robust), Asp (robust). For each protein we computed the fractional fragile amino-acid usage for each of these two groups (count of fragile amino-acids/total count of amino-acids in the group). Finally, for each protein we expressed the normalized fragile amino-acid usage (NFAU) as the average of defined fractions.

### Correlation between transcriptional robustness strategies

Human-mouse orthologs were retrieved using the BioMart tool. In the case of human genes having multiple co-orthologs we retained the longest mouse ortholog (choosing a random protein in the case of length ties). We aligned human-mouse orthologous pairs using CLUSTALW [39] and, using the corresponding CDS, back-translated each alignment to create a codon-based alignment. These alignments were used as input for the yn00 program in the PAML package [40] to estimate K_a_/K_s_ i.e. the ratio of non-synonymous substitutions per non-synonymous site (K_a_) to synonymous substitutions per synonymous site (K_s_). Four groups of human genes of similar selective constraint were constructed based on quartiles of K_a_/K_s_ for all human-mouse orthologs. For each group we calculated the Spearman correlation between normalized fragile codons usage (NFCU) and fragile amino-acid usage (NFAU) metrics.

### Exon-based analysis

We performed an exon-based analysis to compare NFCU in last exons and upstream exons. We considered only genes for which we could be sure that the annotated last exon is the true last exon. Last exons were considered as reliably annotated if they (i) have a CDS sequence that terminates with a STOP codon, (ii) do not have a downstream non-coding exon (and therefore do not have a downstream EJC) and (iii) have an annotated 3′ UTR of at least 100 nts. For each gene having a reliably annotated last exon we assigned the last exon to one group and each remaining exon to a second group (“upstream exons”). We calculated NFCU for each exon using only those codons completely encoded by the exon and compared NFCU in the “last exon” and “upstream exon” groups.

### Determining phylogenetic independence of histone genes

Using the dataset of histone genes retrieved from Ensembl as described above we constructed sequence alignments between all pairs of histone proteins using CLUSTALW [39] and, using the corresponding CDS, back-translated each alignment to create a codon-based alignment. These alignments were used as input for the yn00 program in the PAML package [40] and pairwise sequence divergence was determined by calculating synonymous site divergence (K_s_).

## Supporting Information

Figure S1 The absence of splicing constraints cannot explain the lower fragile codon content of single-exon genes. Normalized fragile codon usage (NFCU) of human single- (S) and multi-exon (M) genes binned by ESE density within the CDS. For each quartile of ESE density (Q1, lowest, to Q4, highest), NFCU for single and multi-exon genes is plotted separately. The width of each bin is proportional to the square root of the number of genes it contains.
(TIF)

Figure S2 Fragile codon usage patterns among human genes are not due to selection for translational accuracy. Normalized fragile codon usage (NFCU) of human single- (S) and multi-exon (M) genes binned by the fraction of translationally optimal codons per gene controlling for GC content (F_op_GC). For each quartile of F_op_GC (Q1, lowest, to Q4, highest), NFCU for single and multi-exon genes is plotted separately. The width of each bin is proportional to the square root of the number of genes it contains.
(TIF)

Figure S3 Fragile codon usage patterns among fly genes are not due to selection for translational accuracy. Normalized fragile codon usage (NFCU) of *Drosophila* single- (S) and multi-exon (M) genes binned by the fraction of translationally optimal codons per gene (F_op_). For each quartile of F_op_ (Q1, lowest, to Q4, highest), NFCU for single and multi-exon genes is plotted separately. The width of each bin is proportional to the square root of the number of genes it contains.
(TIF)

Table S1 Fragile codon usage (FCU) of multi-exon genes and single-exon genes in the human, mouse, and fly genomes.
(DOC)

Text S1 Supporting results.
(DOC)
