# Supplementary figures and images for: Preventing Dangerous Nonsense: Selection for Robustness to Transcriptional Error in Human Genes
Source: PLoS Genet. 2011 Oct 13;7(10):e1002276. doi: 10.1371/journal.pgen.1002276 (PMC3192821; doi:10.1371/journal.pgen.1002276)

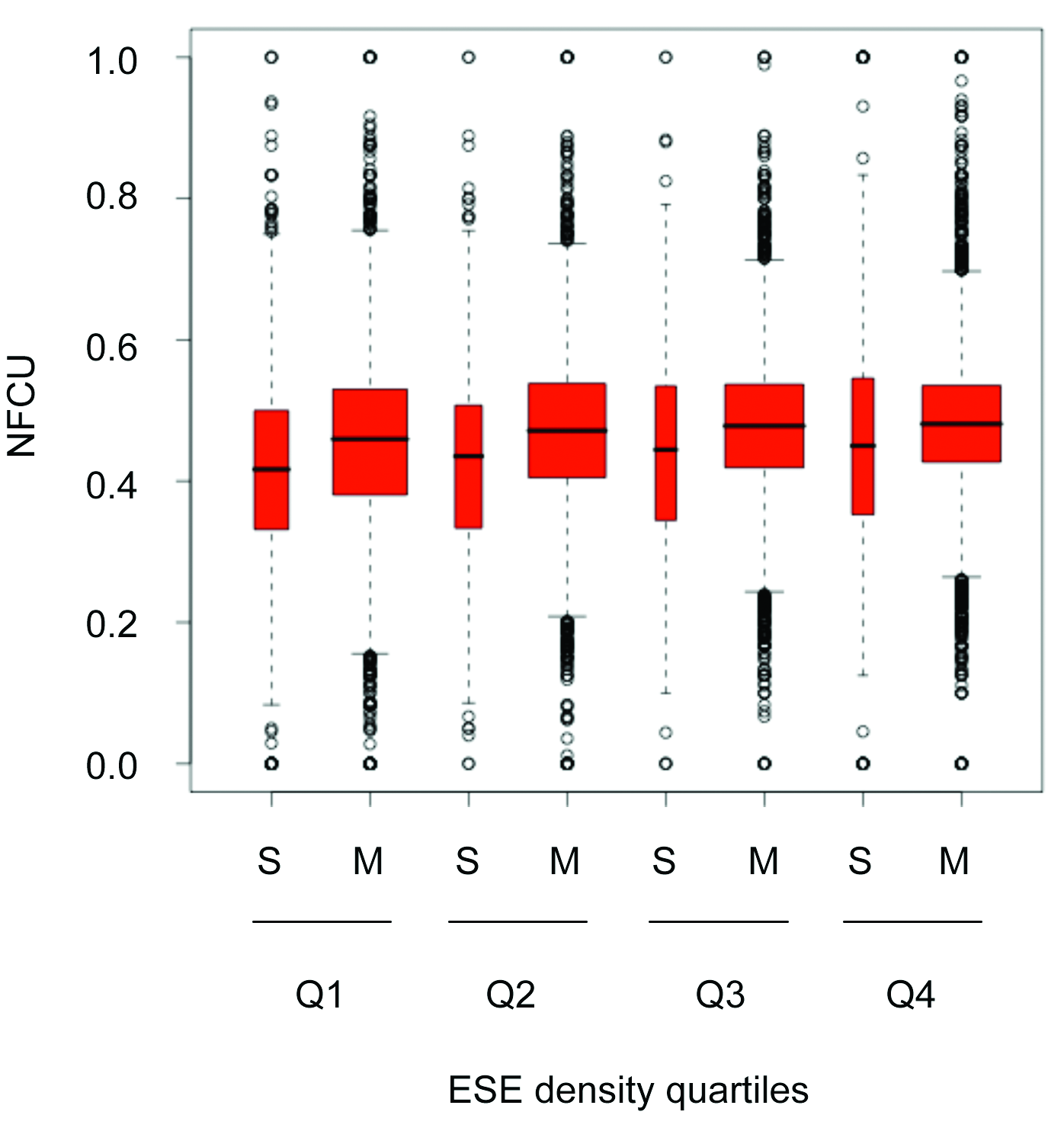

Supplement: Figure S1 — The absence of splicing constraints cannot explain the lower fragile codon content of single-exon genes. Normalized fragile codon usage (NFCU) of human single- (S) and multi-exon (M) genes binned by ESE density within the CDS. For each quartile of ESE density (Q1, lowest, to Q4, highest), NFCU for single and multi-exon genes is plotted separately. The width of each bin is proportional to the square root of the number of genes it contains. (TIF) [file pgen.1002276.s001.tif]

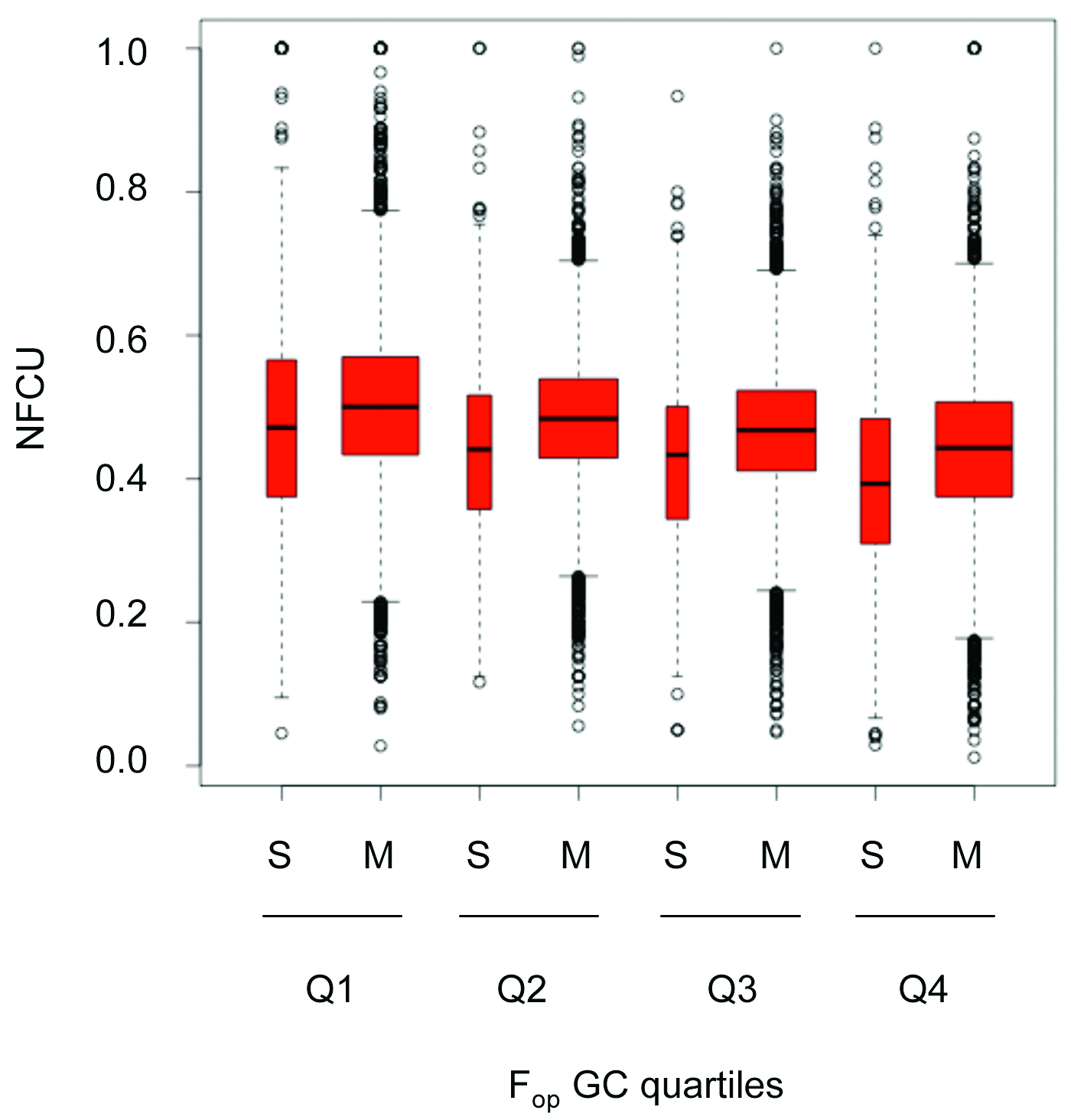

Supplement: Figure S2 — Fragile codon usage patterns among human genes are not due to selection for translational accuracy. Normalized fragile codon usage (NFCU) of human single- (S) and multi-exon (M) genes binned by the fraction of translationally optimal codons per gene controlling for GC content (FopGC). For each quartile of FopGC (Q1, lowest, to Q4, highest), NFCU for single and multi-exon genes is plotted separately. The width of each bin is proportional to the square root of the number of genes it contains. (TIF) [file pgen.1002276.s002.tif]

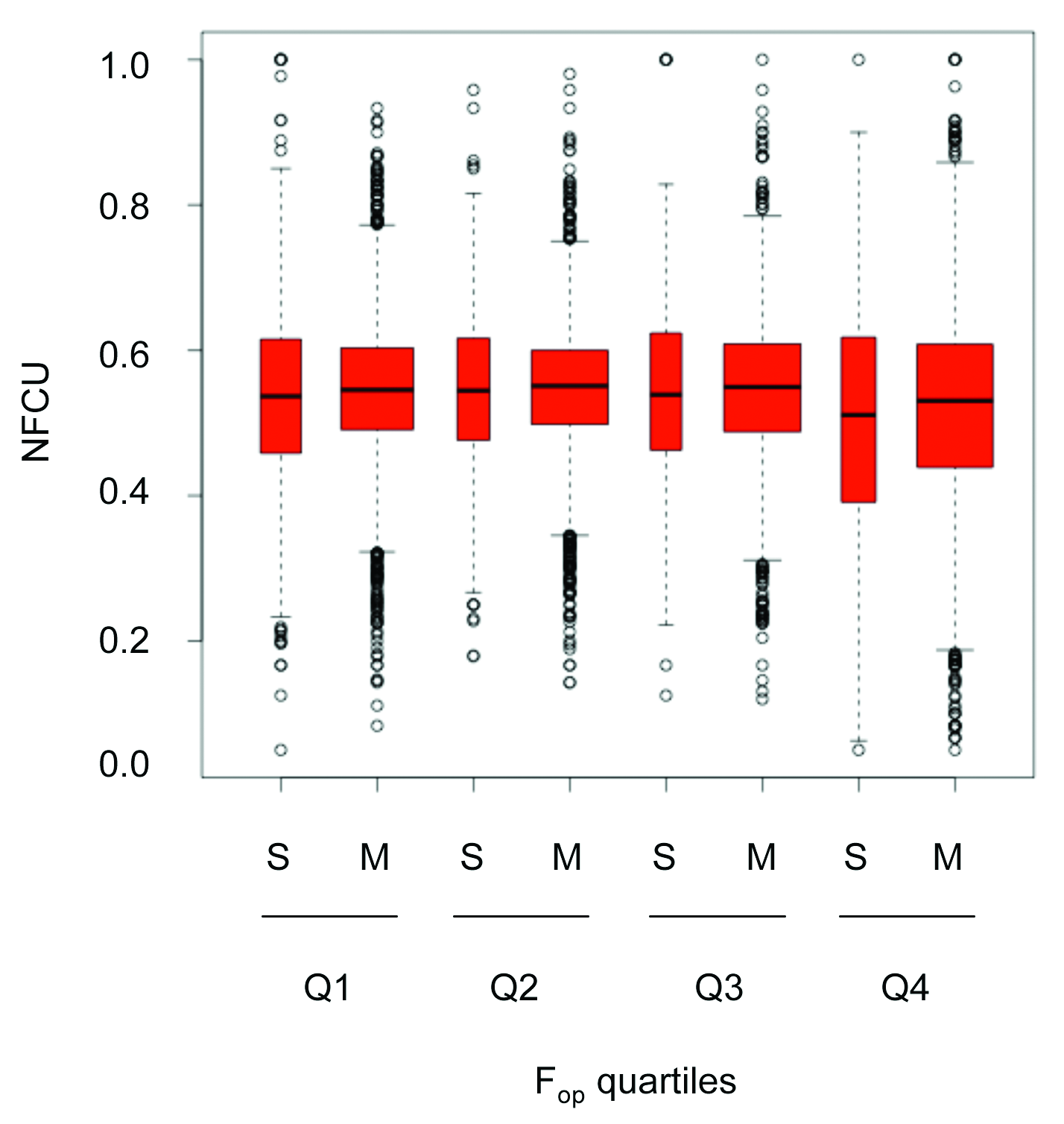

Supplement: Figure S3 — Fragile codon usage patterns among fly genes are not due to selection for translational accuracy. Normalized fragile codon usage (NFCU) of Drosophila single- (S) and multi-exon (M) genes binned by the fraction of translationally optimal codons per gene (Fop). For each quartile of Fop (Q1, lowest, to Q4, highest), NFCU for single and multi-exon genes is plotted separately. The width of each bin is proportional to the square root of the number of genes it contains. (TIF) [file pgen.1002276.s003.tif]
