# Supplementary material for: Preventing Dangerous Nonsense: Selection for Robustness to Transcriptional Error in Human Genes
Source: PLoS Genet. 2011 Oct 13;7(10):e1002276. doi: 10.1371/journal.pgen.1002276 (PMC3192821; doi:10.1371/journal.pgen.1002276)
Supplement: Table S1 — Fragile codon usage (FCU) of multi-exon genes and single-exon genes in the human, mouse, and fly genomes. (DOC) [file pgen.1002276.s004.doc]

**Supplementary Table 1:**

Fragile codon usage (FCU) of multi-exon genes and single-exon genes in the human, mouse and fly genomes.

|  |  | PABP-dependent NMD | EJC-dependent NMD | Genes | FCU | *P*-value | **Ratio** |
| --- | --- | --- | --- | --- | --- | --- | --- |
| **Human genes** |  |  |  |  |  |  |  |
| Multi-exon |  | **+** | **+** | 20573 | 0.31 |  | **1.00** |
|  |  |  |  |  | (0.26– 0.35) |  |  |
| Single-exon: | All | **+/-** | **-** | 2424 | 0.27 | <10^-15^ | **0.88** |
|  |  |  |  |  | (0.23 – 0.32) |  |  |
|  | Non-histone | **+** | **-** | 2367 | 0.27 | <10^-15^ | **0.88** |
|  |  |  |  |  | (0.23 – 0.33) |  |  |
|  | Histone | **-** | **-** | 57 | 0.28 | 0.0003 | **0.91** |
|  |  |  |  |  | (0.26 – 0.30) |  |  |
| **Mouse genes** |  |  |  |  |  |  |  |
| Multi-exon |  | **+** | **+** | 20284 | 0.31 |  | **1.00** |
|  |  |  |  |  | (0.27 – 0.35) |  |  |
| Single-exon: | All | **+/-** | **-** | 3589 | 0.27 | <10^-15^ | **0.87** |
|  |  |  |  |  | (0.23 – 0.33) |  |  |
|  | Non-histone | **+** | **-** | 3540 | 0.27 | <10^-15^ | **0.87** |
|  |  |  |  |  | (0.23 – 0.33) |  |  |
|  | Histone | **-** | **-** | 49 | 0.25 | <10^-12^ | **0.80** |
|  |  |  |  |  | (0.24 – 0.26) |  |  |
| **Fly genes** |  |  |  |  |  |  |  |
| Multi-exon |  | **+** | **-** | 11643 | 0.30 |  | **1.00** |
|  |  |  |  |  | (0.28 – 0.33) |  |  |
| Single-exon: | All | **+/-** | **-** | 2498 | 0.31 | 0.001 | **1.01** |
|  |  |  |  |  | (0.28 – 0.35) |  |  |
|  | Non-histone | **+** | **-** | 2466 | 0.31 | 0.0002 | **1.01** |
|  |  |  |  |  | (0.28 – 0.34) |  |  |
|  | Histone | **-** | **-** | 32 | 0.40 | <10^-9^ | **1.31** |
|  |  |  |  |  | (0.32 – 0.40) |  |  |

Single-exon genes were first treated as a single group to determine the impact of EJC-dependent NMD on fragile codon usage and subsequently subdivided into histone and non-histone genes to consider the impact of PABP-dependent NMD on fragile codon usage. FCU, median (standard deviation) of fragile codon content considering all 61 sense codons; *P*-value, significance of the comparison with multi-exon genes determined by two-sided Wilcoxon-rank sum test that tests for a linear shift in distribution locations; Ratio, median FCU relative to the median FCU for multi-exon genes.
