# Supplementary material for: Preventing Dangerous Nonsense: Selection for Robustness to Transcriptional Error in Human Genes
Source: PLoS Genet. 2011 Oct 13;7(10):e1002276. doi: 10.1371/journal.pgen.1002276 (PMC3192821; doi:10.1371/journal.pgen.1002276)
Supplement: Text S1 — Supporting results. (DOC) [file pgen.1002276.s005.doc]

**Preventing dangerous nonsense: selection for robustness to transcriptional error in human genes**

Brian P. Cusack^1,3,4^, Peter F. Arndt^1^, Laurent Duret^2^, Hugues Roest Crollius^3^

^1^ Max-Planck Institute for Molecular Genetics, Department of Computational Molecular Biology, Berlin, Germany

^2^ Université de Lyon; Université Lyon 1; CNRS; UMR 5558, Laboratoire de

Biométrie et Biologie Evolutive, 43 boulevard du 11 novembre 1918, Villeurbanne

F-69622, France

^3^ Institut de Biologie de l’Ecole Normale Supérieure (IBENS), CNRS UMR8197, INSERM U1024, 75005 Paris, France

^4^ *Corresponding author*. Email: cusack@molgen.mpg.de

**SUPPORTING MATERIAL**

1 Supplementary Results A-G [2](#__RefHeading___Toc173224160)

3 Supplementary References [13](#__RefHeading___Toc173224161)

### 1 Supplementary Results A-G

**A. The inefficiency of NMD in single-exon genes is mitigated through a general avoidance of fragile codons**

We compared 2424 single-exon genes to 20573 multiple-exon genes in the human genome with respect to their fractional fragile codon usage (FCU) calculated over all 61 sense codons. We found that human single-exon genes (median FCU = 0.27) show a highly significant (*p* < 10^-15^, Wilcoxon rank sum test) 12% reduction in their usage of fragile codons compared to multi-exon genes (median FCU = 0.31; Supplementary Table 1). Similarly, in mouse, we saw a 13% (*p* < 10^-15^, Wilcoxon rank sum test) depletion of fragile codons in single-exon genes (median FCU = 0.27) compared to multi-exon genes (median FCU = 0.31). As a negative control we repeated the analysis of single-exon and multi-exon genes in *Drosophila melanogaster* where the efficiency of NMD is expected to be similar in both types of genes. Notably in the fly genome, we found no depletion of fragile codons in single-exon (n = 2498, median FCU = 0.31) compared to multi-exon genes (n = 11643, median FCU = 0.30; Supplementary Table 1).

**B. The absence of splicing constraints cannot explain the lower fragile codon content of single-exon genes**

To test whether differences in splicing-related constraints might explain our result we separately calculated the tendency for fragile and robust codons to be encoded by exonic splicing enhancer (ESE) hexamers by employing the hexamer preference index (HPI) metric described by Parmley et al [1] and using a collection of 443 hexamers determined as likely to function as splice enhancers according to the RESCUE-ESE protocol. The HPI for fragile and robust codons is 1.16 and -2.85 respectively. This indicates that ESE-hexamers that are randomly phased with respect to the coding sequence tend to encode fragile codons but tend not to encode robust codons. In addition we calculated the density of these hexamers in the CDS of single and multi-exon genes. As expected, we found that ESEs are depleted in single exon genes: in human single-exon genes 27% of coding nucleotides overlap an ESE-hexamer whereas in multi-exon genes this proportion is 31%.

This difference in ESE-density could explain the difference in fragile codon usage between single and multi-exon genes. To control for this possibility we compared NFCU between human single and multi-exon genes binned by ESE density (fig. S1). We found that fragile codons are significantly (*p* < 10^-4^, Wilcoxon rank-sum test) depleted in single-exon genes independently of ESE-density (ratios of median NFCU in single-exon genes relative to median NFCU in multi-exon genes for ESE-density bins Q1-Q4: 0.91, 0.92, 0.93, 0.94, respectively). This allows us to rule-out the possibility that the difference in fragile codon usage between single and multi-exon genes is a simple artifact of differences in splicing constraints.

**C. The lower fragile codon content of single-exon genes cannot be explained by selection for translationally optimal codons**

We first tested whether there is an association among codons between fragility with respect to nonsense errors and translational optimality in human or fly. In human, five out of 18 (28%) fragile codons are translationally optimal, whereas 14 out of 43 (33%) robust codons are translationally optimal (*p* = 0.95, χ^2^ test). In fly, five out of 18 (28%) fragile codons are translationally optimal, whereas 11 out of 43 (26%) robust codons are translationally optimal (*p* = 0.89, χ^2^ test). Considering only the 11 codons used in the NFCU metric, in human none of the five fragile codons are translationally optimal, compared with one out of six robust codons. Similarly, in fly, one out of five fragile codons are translationally optimal, compared with one out of six robust codons.

To control for the possibility that there is an association among genes between transcriptional robustness and selection for translational accuracy we repeated the analysis of fragile codon content in human and fly by binning all genes with respect to the fraction of optimal codons per gene (F_op_). Specifically, we assigned *Drosophila* genes to bins defined by the quartiles of the distribution of F_op_ (fractional optimal codon usage) [2] for all fly genes. Fly optimal codons were as defined in [2]. The modified F_op_GC metric was used in human to control for variation in GC-content due to the isochore effect as described in [3] and using human optimal codons defined in [4]. For each bin we then compared FCU and NFCU for single and multi-exon genes that have the same average usage of translationally optimal codons.

In human, we found that controlling for translational selection does not influence our results. Considering the NFCU metric (i.e. controlling for amino acid usage and nucleotide composition), we found that fragile codons are significantly (*p* < 10^-8^) depleted in single-exon genes independently of translational selection (ratios of median NFCU in single-exon genes relative to median NFCU in multi-exon genes for F_op_GC quartiles Q1-Q4: 0.94, 0.91, 0.93, 0.89, respectively) (fig. S2). Similarly, considering the non-normalized FCU metric (which takes advantage of all sense codons), we found that fragile codons are significantly (*p* < 10^-15^) depleted in human single-exon genes independently of translational selection (ratios of median FCU in single-exon genes relative to median FCU in multi-exon genes for F_op_GC quartiles Q1-Q4: 0.87, 0.87, 0.88, 0.91, respectively).

Equally, in fly, we found that our results remained unchanged once translational selection is controlled for. Considering the NFCU metric, we found that the fragile codon content of single-exon genes continues to show only a modest depletion (*p* > 0.01) relative to multi-exon genes (ratios of median NFCU in single-exon genes relative to median NFCU in multi-exon genes for F_op_ quartiles Q1-Q4: 0.98, 0.99, 0.98, 0.96, respectively) (fig. S3). Similarly, considering the non-normalized FCU metric following our control for translational selection, we found that fragile codons are not depleted in single-exon genes (ratios of median FCU in single-exon genes relative to median FCU in multi-exon genes for F_op_ quartiles Q1-Q4: 1.01, 1.01, 0.99, 1.02, respectively).

**D. Among multi-exon genes fragile codon content is dependent on gene structure**

The fact that only PTCs lying more than ~50-55 nts upstream of the last Exon Junction Complex (EJC) are thought to be detected by the EJC-dependent NMD pathway suggests that multi-exon genes whose coding sequence is completely contained within the last exon should be particularly robust to nonsense transcriptional errors.

We considered fragile codon usage in two distinct groups of human multi-exon genes: those for which only the last exon is coding (with all upstream exons encoding the 5’ UTR) and those having at least two coding exons (i.e. where the last exon is coding and where at least one upstream coding exon exists). The coding regions of genes in the former group are completely invisible to EJC-dependent NMD and are served only by PABP-dependent NMD (i.e. are “NMD-compromised”). They therefore should be subject to strong selective constraint to maintain their transcriptional robustness. In accordance with this expectation, in human, we observed a significant 8% depletion of fragile codons (Wilcoxon rank-sum test *p* < 10^-15^) among these multi-exon genes (n = 1034, median NFCU = 0.437) compared to multi-exon genes having at least one upstream coding exon (n = 19529, median NFCU = 0.475). Notably, this difference persists when we control for differences in CDS length and ESE density between these gene categories in human (data not shown). Interestingly, human multi-exon genes whose last exon is the only coding exon have a fragile codon content similar to that of single-exon genes (median NFCU = 0.43; main text).

Similarly, in mouse, we saw a significant 11% depletion of fragile codons (Wilcoxon rank-sum test *p* < 10^-15^) in multi-exon genes where only the last exon is coding (n = 1166, median NFCU = 0.417) compared to multi-exon genes having at least one upstream coding exon (n = 19097, median NFCU = 0.471).

As a negative control we repeated this analysis using *Drosophila* genes and saw a more modest 3% reduction in fragile codon content among multi-exon genes whose CDS is encoded exclusively by the last exon (n = 429, NFCU = 0.527) compared with multi-exon genes whose CDS is encoded by at least one upstream exon (n = 11214; NFCU = 0.546; Wilcoxon rank-sum test *p* = 0.015). However, in contrast to the analysis of human genes, when differences in CDS length between these two fly gene sets were controlled for we saw no consistent pattern of depletion of NFCU among the former genes (data not shown).

**E. Differences in splicing constraints do not explain reduced fragile codon content of last exons**

Although the depletion of fragile codons in last exons of multi-exon genes suggests that these exons are robust to nonsense transcriptional errors (main text), splicing-related constraints have a confounding influence similar to that described for the comparison of single-exon and multi-exon genes. We noticed that among human multi-exon genes, the density of exonic splicing enhancers (ESEs) in last exons is significantly lower than that of upstream exons (median ESE density 0.29 and 0.36 respectively, Wilcoxon rank-sum test *p* < 10^-15^). This suggests that the depletion of fragile exons in last exons is due to a reduction in splicing constraints on the final exon of transcripts rather than reflecting a requirement for transcriptional robustness. To control for this effect, we partitioned codons in each exon into those that overlap ESEs and those external to ESEs and recalculated NFCU for each partition.

Among codons overlapping ESEs we observed a significant 2% depletion (Wilcoxon rank-sum test *p* < 10^-8^) in mean fragile codon density in last exons (N= 10412, mean NFCU = 0.614) compared to upstream exons (N= 91140, mean NFCU = 0.625) but no depletion in median fragile codon density. In contrast, for codons external to ESEs we observed an insignificant 3% depletion (Wilcoxon rank-sum test *p* = 0.97) in mean fragile codon density in last exons (N= 11587, mean NFCU = 0.335) compared to upstream exons (N= 104576, mean NFCU = 0.344) but again no depletion in median fragile codon density.

**F. The lower fragile codon content of single-exon genes cannot be explained by transcription-associated mutational bias**

Transcription-associated mutational bias (TAMB) could systematically bias codon usage and thus might impact on the relative usage of fragile and robust codons. Two biases, one acting globally and one acting locally within transcribed regions, are known to exist and both affect the ratio of A to G at synonymous sites on the coding strand [5,6]. The global mutational asymmetry creates an excess of G+T over A+C on the coding strand presumably as a consequence of biases in the process of transcription-coupled repair in germline cells [5]. The second mutational asymmetry creates an excess of C to T over G to A transitions on the coding strand and is localized to the first 1-2kb downstream of the TSS [6]. This bias also creates an excess of G+T over A+C on the coding strand. Both mutational patterns can affect the ratio of A to G at synonymous sites.

Conceivably, if these patterns differentially affect single-exon and multi-exon genes then the difference in fragile codon density of these genes might have a neutral mutational explanation rather than reflecting a difference in transcriptional robustness. This could occur in two ways. First, the localized mutational pattern has a disproportionate impact on codon evolution in single-exon genes since the fraction of the coding sequence lying within 1-2kb of the TSS is larger for single-exon than for multi-exon genes. Second, since both mutational asymmetries are heritable only when a gene is expressed in the germline, a difference in the frequency of germline expression between single-exon and multi-exon genes could explain our observation.

Taken together these patterns of TAMB can confound our measures of transcriptional robustness by affecting the ratio A/G at synonymous sites. However, we can disentangle the influence of TAMB and transcriptional robustness by calculating this ratio separately for synonymous changes that do and do not affect codon fragility. For human single and multi-exon genes we computed the ratio SGA/SGG (i.e. fragile codon/robust codon) and compared it to STA/STG (i.e. robust codon/robust codon), where S is C or G. In principle both SGA/SGG and STA/STG should be affected by TAMB (and the isochore effect), but only SGA/SGG is affected by selective constraint on transcriptional robustness. Moreover, both measures consider only pairs of synonymous codons and thus are not confounded by amino-acid level selection.

We saw no significant difference in the ratio STA/STG between single-exon genes (median STA/STG = 0.208, n = 2153) and multi-exon genes (median STA/STG = 0.195, n = 19918) (*p* = 0.64, Wilcoxon rank-sum test). By contrast, the ratio SGA/SGG showed a 14% reduction in single-exon (median SGA/SGG = 0.65, n = 2153) compared to multi-exon genes (median SGA/SGG = 0.75, n = 19918)
(*p* < 10^-15^, Wilcoxon rank-sum test). This suggests that the influence of TAMB (combined with the isochore effect) does not explain differences in the fragile codon content of single and multi-exon genes. Moreover, its effect is likely to underestimate the impact of selection for transcriptional robustness since we observe a greater depletion (14%) of fragile codons in single-exon genes than that seen when we do not control for the effect of TAMB (8% depletion in NFCU).

**G. Accounting for gene prediction and phylogenetic artifacts**

We sought to exclude two potential technical artifacts as explanations for the observed differences in fragile codon usage between single-exon and multi-exon genes.

First, we accounted for the possibility that false positive gene predictions contribute to the difference in fragile codon density between single-exon and multi-exon genes. To this end we restricted the analysis to human genes that have an identifiable ortholog in mouse and are therefore more reliable gene predictions. Using the BioMart tool we retrieved a set of human genes having mouse orthologs. Strikingly, after applying this filter 922 single-exon genes and 16553 multi-exon genes remained (38% and 80% of the total gene sets, respectively). The lower retention rate of single-exon genes suggests that a greater fraction of these genes are either (i) false positive gene predictions, (ii) lineage-specific genes or (iii) fast-evolving genes without an identifiable ortholog in mouse. A comparison of the two groups of reliable human gene predictions revealed essentially the same depletion of fragile codons (7%; *p* < 10^-15^, Wilcoxon rank-sum test) in single-exon genes (n = 922, median NFCU = 0.44) compared to multi-exon genes (n = 16553, median NFCU = 0.48) as that seen in the full dataset (8%), indicating that gene annotation artifacts do not explain our result.

Second, we assessed whether the difference in fragile codon density between human single and multi-exon genes could be a consequence of bias introduced by the inclusion of multiple observations from large gene families. For example, a large family of closely related single-exon paralogs (e.g. olfactory receptor genes) could contribute multiple datapoints that are not phylogenetically independent thus skewing our estimate of the average fragile codon density of single-exon genes.

We used a conservative approach to ensure that each datapoint in our dataset of human genes is phylogenetically independent with respect to fragile codon usage as measured by NFCU. First we identified duplicated genes by retrieving a list of genes having at least one human paralog from Ensembl using the BioMart tool. Then we subtracted all these duplicated genes from our dataset to generate a set of human singleton genes. Since the NFCU metric measures the effect of transcriptional robustness on synonymous codon choice this approach is highly conservative. This is due to the fact that we removed all gene duplicates including those that are sufficiently old that synonymous divergence has saturated thus rendering their measurements of NFCU phylogenetically independent. We can therefore be confident that the remaining single exon and multi-exon singleton genes are phylogenetically independent observations that are representative of fragile codon usage in their respective categories.

This reduced singleton dataset consists of 1193 single exon genes and 8414 multi-exon genes. We observed the same depletion of fragile codons (8%, *p* < 10^-15^, Wilcoxon rank-sum test) in single-exon genes (n = 1193, median NFCU = 0.43) compared to multi-exon genes (n =8414, median NFCU = 0.47) as that seen in the full dataset (8%), implying that our observation is not biased by the occurrence of large gene families.

Similarly, the inclusion of multiple histone genes with a high degree of sequence similarity in our dataset might introduce a bias due to phylogenetic dependencies in a manner similar to that described above for other single-exon genes. Dependencies between histone genes with respect to estimates of transcriptional robustness can arise as a consequence of insufficient sequence divergence due to either recent gene duplication or to gene conversion between more distantly related duplicates. We controlled for this effect using a conservative approach that retains only histone genes that are phylogenetically independent at the level of synonymous site divergence. We determined synonymous site divergence (K_s_) between all pairs of histone genes in the human dataset and both members of any pair having K_s_ < 1 were removed from our dataset. This has the effect of ensuring that any duplicates retained have undergone, on average, at least one substitution at each synonymous site since their divergence and are therefore independent with respect to NFCU.

The filtered dataset of phylogenetically independent histone genes consists of 34 genes with a median NFCU of 0.33. This represents a depletion of fragile codons of 24% compared to other single-exon genes (*p* = 0.001, Wilcoxon rank-sum test) and of 31% compared to multi-exon genes (*p* < 10^-6^, Wilcoxon rank-sum test). Since our results were essentially unchanged by this control, phylogenetic dependencies among histone genes do not bias our results.

### 3 Supplementary References

1. Parmley JL, Urrutia AO, Potrzebowski L, Kaessmann H, Hurst LD (2007) Splicing and the evolution of proteins in mammals. PLoS Biol 5: e14.

2. Duret L, Mouchiroud D (1999) Expression pattern and, surprisingly, gene length shape codon usage in *Caenorhabditis*, *Drosophila*, and *Arabidopsis*. Proc Natl Acad Sci U S A 96: 4482-4487.

3. Drummond DA, Wilke CO (2008) Mistranslation-induced protein misfolding as a dominant constraint on coding-sequence evolution. Cell 134: 341-352.

4. Comeron JM (2004) Selective and mutational patterns associated with gene expression in humans: influences on synonymous composition and intron presence. Genetics 167: 1293-1304.

5. Green P, Ewing B, Miller W, Thomas PJ, Green ED (2003) Transcription-associated mutational asymmetry in mammalian evolution. Nat Genet 33: 514-517.

6. Polak P, Arndt PF (2008) Transcription induces strand-specific mutations at the 5' end of human genes. Genome Res 18: 1216-1223.
